# Beneficial Metabolic Effect of a Nutraceuticals Combination (Monacolin K, Yeasted Red Rice, Polyphenolic Extract of Annurca Apple and Berberine) on Acquired Hypercholesterolemia: A Prospective Analysis

**DOI:** 10.3390/metabo11040223

**Published:** 2021-04-06

**Authors:** Roberta D’Assante, Mariarosaria De Luca, Sergio Ferraro, Andrea Ferraro, Antonio Ruvolo, Francesco Natale, Pietro Sotgiu, Maurizio Petitto, Romolo Rizzo, Umberto De Maria, Luigi Liguori, Gianluigi Gentile, Paola Ragucci, Vittorio Donadio, Valeria Valente, Antonio Cittadini

**Affiliations:** 1 Department of Translational Medical Sciences, Federico II University of Naples, 80100 Naples, Italy; roberta.dassante@outlook.it (R.D.); deluca.mrs@libero.it (M.D.L.); valeriaelettravalente@gmail.com (V.V.); 2 A.O.R.N. Ospedali dei Colli–CTO, 80100 Naples, Italy; srg.ferraro@virgilio.it (S.F.); andreaferraro@laboratorisander.it (A.F.); ruvantonio@libero.it (A.R.); tsuru31@hotmail.it (R.R.); 3 A.O.R.N. Ospedali dei Colli–Monaldi, 80100 Naples, Italy; natalefrancesco@hotmail.com; 4 Casa di Cura Maria Rosaria di Pompei, 80100 Naples, Italy; p.sotgiu_md@libero.it; 5 Santa Maria della Pietà Camilliani Hospital, 80100 Naples, Italy; maurizio.petitto@unina.it; 6 Centro Gal.Ma.Ca, 80100 Naples, Italy; dott.umbertodemaria@hotmail.it; 7 General Practitioner, Via A. Rocco 13, 80100 Naples, Italy; drliguoriluigi@libero.it; 8 Distretto 33, ASL Napoli 1, 80100 Naples, Italy; giangigentile@libero.it (G.G.); paoladoc@tiscali.it (P.R.); 9 Sander Laboratories, 80100 Naples, Italy; info@laboratorisander.it

**Keywords:** hypercholesterolemia, triglyceride, nutraceutical, Melasterol, annurca apple, red yest rice, berberine

## Abstract

Hypercholesterolemia represents a serious public health problem as it significantly increases the risk of developing cardiovascular diseases. Its treatment with statin is limited by costs, side effects, and drugs interactions. Nutraceuticals appear to have an important metabolic effect on cholesterol reduction as well as on body weight and glycemia. The aim of this study was to evaluate the effect of a nutraceutical combination (Melasterol) in eighty-seven patients with acquired hypercholesterolemia. Clinically relevant parameters were collected at baseline and after three and six months of Melasterol treatment, one tablet per day. The primary endpoint was the change in cholesterol and triglyceride levels. Six months of treatment resulted in a 19.2% decrease in total cholesterol, accompanied by a 19.8% decrease in low-density lipoprotein (LDL) and a 23% reduction in triglycerides (*p* < 0.001) but not in high-density lipoprotein (HDL) levels (*p* > 0.05). These results were paralleled by a significative blood glucose (108.3 ± 21.3 vs. 98.4 ± 18.6 mg/dL *p* < 0.001) and body mass index (BMI) reduction (27.8 ± 4.4 vs. 27.0 ± 4.2 mg/dL, *p* < 0.001). A subgroup of 12 patients performed flow-mediated dilation, with values increasing by 1.8% (*p* < 0.05). No significant side effects were reported. Besides its cholesterol-lowering effect, Melasterol was associated with a significant improvement in other relevant metabolic parameters such as BMI and glycemia.

## 1. Introduction

Hypercholesterolemia represents a serious public health problem as it significantly increases the incidence of cardiovascular diseases (CVD), the first cause of morbidity and mortality worldwide [1,2]. Acquired hypercholesterolemia is characterized by increased low-density lipoprotein (LDL) and reduced high-density lipoprotein (HDL) cholesterol levels [3,4], which should be analyzed every time cardiovascular risk is assessed.

The European Society of Cardiology (ESC) guidelines on the management of dyslipidemia suggest intervention strategies as a function of total cardiovascular risk and lipid profile. Lifestyle modifications are suggested in all patients with dyslipidemia, regardless of risk class [5]. They are based on the consumption of healthy functional foods and/or dietary supplements [5,6,7]. Additionally, the therapeutic approach with drugs able to reduce cholesterol levels, such as statins, is the most common method for the treatment of hypercholesterolemia. However, the use of statins is limited by their relevant interactions with other drugs and side effects, so that many researchers are investigating effective and safer alternative treatments [5,6,7,8]. In this context, nutraceuticals represent an emerging trend in healthcare for their metabolic effects on cholesterol and ancillary parameters such as body weight and glycemia. Nutraceuticals should be considered in monotherapy in patients with mild–moderate hypercholesterolemia and low–moderate cardiovascular risk; in monotherapy and in combination in case of lack of any other possibilities to achieve the LDL cholesterol (LDL-C) targets with statin and non-statin agents, including in high-risk patients with complete or partial statin intolerance who have not reached LDL-C goal with tolerable statin therapy and/or non-statin therapy [5,6,7].

A growing number of natural substances with different cholesterol-lowering efficacy (e.g., phytosterols, soya, fermented red rice, and berberine) have been proposed [9]. Monacolin K contributes to the reduction of circulating and liver cholesterol levels by increasing liver endocytosis of LDL-C and reducing liver biosynthesis of cholesterol through the inhibition of 3-hydroxy-3-methyl-glutaryl-CoA (HMG-CoA) reductase [10,11,12]. Berberine, a plant-derived alkaloid, reduces proprotein convertase subtilisin/kexin type 9 (PCSK9) expression and, consequently, LDL-receptor (LDLR) degradation, promoting LDL bloodstream clearance [13,14,15]. Other studies have documented a reduction of triglyceride synthesis, an improvement in endothelial function, and important cardiac inotropic and antiarrhythmic effects [16,17,18].

Many dietary fruits and vegetables also have hypolipidemic properties and beneficial effects against cardiovascular diseases, cancer, diabetes, lung disorders, Alzheimer’s dementia, and other degenerative diseases [19]. In particular, the scientific community is currently focusing attention on the antioxidant power of apples and their derivatives, due to the presence of polyphenols, which make up 0.01% to 1% of their fresh weight [20]. Furthermore, annurca apples reduce total cholesterol levels, increase HDL levels, and exert antioxidative actions [21,22]. The consumption of apples alone cannot ameliorate the cardiovascular risk, but the high amounts of polyphenols contained in nutraceuticals could exert a significant healthy beneficial effect.

In this context, Melasterol has been formulated to take advantage of the presence and the synergistic hypolipidemic action of all these components. The aim of this study was to investigate the effects of a six-month Melasterol treatment on lipid metabolism in a population of hypercholesterolemic patients at low–moderate cardiovascular risk.

## 2. Results

Eighty-seven patients were enrolled in the study. The main anthropometric characteristics and blood chemistry parameters of the study population are reported in Table 1. BMI: Body Mass Index. HDL: High Density Lipoprotein. LDL: Low Density Lipoprotein.

**Figure 1:**
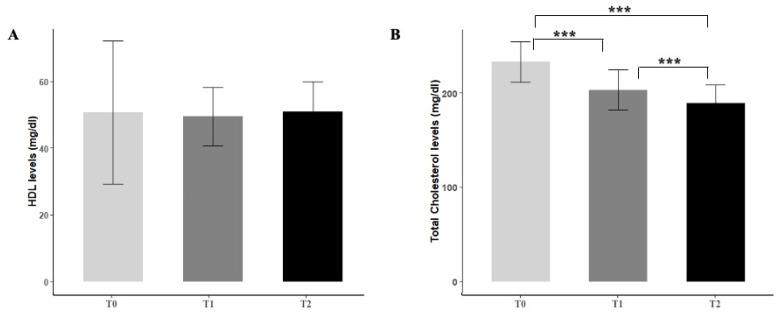

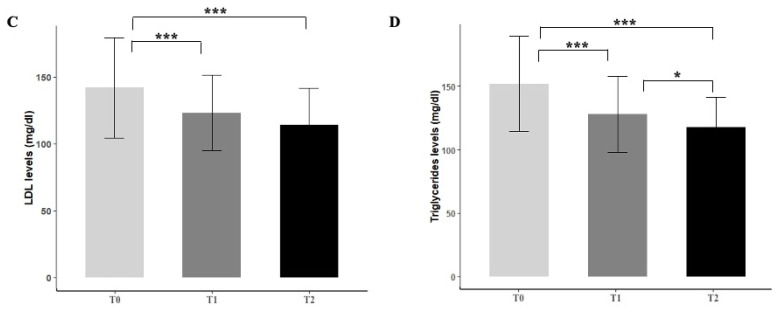
Effect of Melasterol administration on HDL (**A**), total cholesterol (**B**), LDL (**C**), and triglycerides levels (**D**) after 3 months (T1) and 6 months of treatment (T2), respectively. Values are represented as mean ± standard deviation. * *p <* 0.05, *** *p <* 0.001.

Six months of Melasterol treatment resulted in an overall significant reduction in the parameters considered. No relevant effects were observed about HDL levels (Figure 1A). When compared with baseline, a 19.2% decrease in total cholesterol levels at 6 months was found (232.8 ± 21.3 vs. 189 ± 19.4, *p* < 0.001) (Figure 1B), together with a 19.8% decrease in LDL levels (141.8 ± 37.3 vs. 114 ± 27.5, *p* < 0.001) (Figure 1C). The most marked reduction among all the parameters considered regarded triglycerides levels, with a 23% reduction (*p* < 0.001) (Figure 1D).

In addition, a significative blood glucose level (T0 108.3 ± 21.3 mg/dL vs. T2 98.4 ± 18.6 mg/dL, *p* < 0.001) and BMI reduction (T0 27.8 ± 4.4 mg/dL vs. T2 ± 27.0 ± 4.2, *p* < 0.001) were observed.

**Figure 2:**
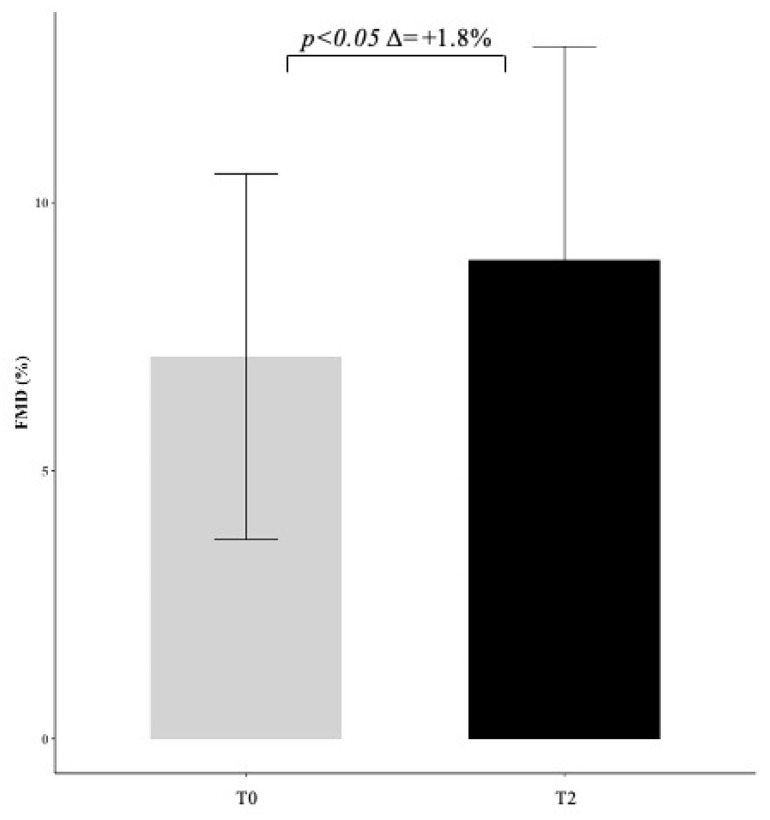
Effect of Melasterol administration on flow-mediated dilation (FMD) represented as delta change (+1.8) between baseline (T0) and follow-up (T2) measurements.

A subgroup of 12 patients performed flow-mediated dilation (FMD), with values increasing from 7.1 ± 3.5 to 8.9 ± 4.0 (*p* < 0.05) after 6 months of treatment with Melasterol (Figure 2). Melasterol treatment was well tolerated by all patients, who did not report side effects.

## 3. Discussion

Hypercholesterolemia is one of the main factors of the global cardiovascular burden and requires specific lifestyle and pharmacological interventions. Nutraceuticals with different cholesterol-lowering efficacy have been proposed with the aim to identify new alternative strategies to the use of statins. In our study, we collect and analyze data regarding the effect of Melasterol therapy on the lipid profile of hypercholesterolemic patients at low–moderate cardiovascular risk. Results are intriguing: when compared with baseline values, total cholesterol, LDL, and triglycerides levels resulted significantly reduced after 6 months of therapy. An upward trend in HDL cholesterol was also observed with no statistical differences with baseline. Our results are consistent with previous research focused on the single components of Melasterol (red yeast rice, berberine, and annurca apple).

It is certainly important to underline that the reduction in LDL and total cholesterol levels was already noticeable after only three months of treatment.

Several studies demonstrated that red yeast rice intake reduces total cholesterol from 16% to 31% and LDL cholesterol from 22% to 32%, lowering the risk of coronary artery heart disease and mortality [23,24]. Monacolin K, a fermented product of rice, is the main substance responsible for the lipid-lowering effect, inhibiting HMG-CoA reductase. Moreover, red yeast rice contains phytosterols, which reduce intestinal cholesterol absorption [9].

Berberine is a nutraceutical extracted from the root of the Berberis plant, with a beneficial effect on LDL cholesterol levels, inducing a reduction ranging from 10 to 20% [25]. Multiple mechanisms of action have been proposed: the increased expression of the hepatocyte’s LDL receptor through a post-transcriptional mechanism that stabilizes the LDLR mRNA, the increased transcriptional activity of its promoter, the reduction of the transcription of PCSK9 gene, and the reduction of cholesterol synthesis through the inhibition of HMG-CoA reductase [13].

Annurca apple contains procyanidin B2, which exerts anti-atherosclerotic beneficial effects as demonstrated by the reduction of total and LDL cholesterol by acting with a β-cyclodextrin-like mechanism, accumulating in the intestinal lumen where they inhibit the cellular cholesterol uptake by building micelle-like complexes in which cholesterol is incorporated and increasing HDL cholesterol plasma levels by binding to these circulating molecules [21,26,27].

A recent randomized clinical trial performed to test a novel Annurca apple-based nutraceutical formulation effect on lipid profile showed a reduction in the mean values of LDL-C by 37.5% and an increase in HDL-C by 49.2% [26].

In the present study, these effects were all confirmed except for HDL levels, which showed only a not significant improvement. The 19% mean reduction of cholesterol levels observed in our study is comparable to the effects obtained with different statins at different dosages [28]. Statins reduce cholesterol by acting on cholesterol biosynthesis in the liver. In particular, the competitive block of the active site of HMG-CoA reductase prevents substrate access and the activation of the mevalonate pathway. Statins also act as modulators of the entire lipid profile by reducing the synthesis and secretion of triglyceride-rich lipoproteins and increasing the production of receptors for apolipoproteins B/E, by inhibiting the hepatic synthesis of apolipoprotein B-100 [29]. Among the statins, the lowest mean percentage LDL-C reduction can be obtained with simvastatin 10 mg, lovastatin 20 mg, or pravastatin 10–20 mg (low intensity, <30%). The highest LDL-C reduction can be achieved with rosuvastatin 20 and 40 mg and with atorvastatin 80 mg (high intensity > 50%). According to our results, Melasterol mean LDL reduction is comparable to the effect obtained with simvastatin or with low doses of atorvastatin and rosuvastatin [30].

These findings have several implications: Melasterol could exert healthy benefits and be advised for its cholesterol-lowering activity in some particular categories of patients, such as patients with mild/moderate hypercholesterolemia and low/moderate cardiovascular risk, patients assuming drug therapy with not-optimal control, or patients who refer intolerance to multiple statins [5,6,7,8].

Preparations containing multiple nutraceutical substances can amplify the results obtained on total and LDL cholesterol by individual molecules but also achieve additive effects on metabolism. Indeed, an important result of our study is the significant reduction of glycemia. Accordingly, BMI was significantly reduced. These results suggest a potential role of Melasterol in patients with mild hypercholesterolemia and obesity and/or impaired glucose metabolism. In fact, all nondiabetic patients with impaired fasting glucose at the enrollment had blood glycemia <100 mg/dL after 6 months and reduced their body weight.

Finally, in a subgroup of 12 patients who performed FMD before and after 6 months of Melasterol treatment, a significant improvement was observed, suggesting a favorable effect on endothelium, whose dysfunction is a cornerstone in the pathogenesis of cardiovascular disease. Melasterol could exert a protective role, improving both lipid profile and vascular homeostasis. The collection of these data was intended as a preliminary analysis.

This hypothesis should be confirmed by studies specifically designed and carried out on a larger sample of patients in order to obtain an overall evaluation of vascular effects.

## 4. Materials and Methods

### 4.1. Study Design, Study Participants

This is a prospective observational study conducted on patients with acquired hypercholesterolemia undergoing one tablet daily of Melasterol for six months, as indicated by the respective General Practitioner. Melasterol is a nutraceutical compound containing monacolin K (5 mg), berberine (500 mg), and polyphenolic extract of Annurca apple (400 mg).

Its intake was suggested before or after the main meal (preferably in the evening) as the polyphenolic extract of Annurca apple reduces cholesterol absorption. The rationale about dose choice relies on the fact that monacolin K can give statin-like side effects, plus the maximum dosage of berberine for a dietary supplement is 500 mg per day. Eligibility criteria were as follows: age > 18 years; diagnosis of hypercholesterolemia according to ESC/EAS (European Atherosclerosis Society) guidelines [5]. Patients with comorbidities were included, provided their lifestyle and treatment did not change during the six-month follow-up. Patients were asked to keep their dietary habits and physical activity unchanged throughout the entire study.

Exclusion criteria were as follows: treatment with other hypolipidemic drugs in the six weeks before baseline assessment; indications to initiate statin according to patient’s cardiovascular risk; intolerance to any component of Melasterol.

Eighty-seven patients meeting the eligibility criteria, according to the indication of the respective General Practitioner, were enrolled and then followed for six months in several Outpatient Units in Campania, Italy. Relevant clinical data were collected anonymously at the basal examination (T0), after three months (T1), and after six months (T2) of treatment and then referred to a single statistical center, the Internal Medicine of the AOU Federico II of Naples. Patients were evaluated with medical history and physical examination; anthropometric parameters were measured. Total cholesterol, HDL, LDL, triglycerides, and glycaemia results were reported by the patient and anonymously collected by physicians.

Data regarding FMD before and after Melasterol treatment were available for 12 patients and were also collected. FMD had been carried out according to current guidelines [31]. FMD was performed only in a subgroup of patients who referred to medical centers where the technique was available.

The study protocol was approved by the Ethics Committee of the Federico Ⅱ University of Naples (Prot. n. 344/20).

The primary endpoint of the study was the change in total cholesterol, LDL, HDL, and triglyceride levels, following the administration of Melasterol, one tablet daily for six months. The secondary endpoint was the modifications of other parameters, such as body weight and glycemia.

### 4.2. Statistical Analysis

Normally distributed continuous variables were expressed as mean ± standard deviation (SD). All variables were tested for normal distribution using the Kolmogorov–Smirnov test. Normally distributed variables were compared using the two-sided, unpaired Student’s *t*-test, assuming unequal variance or one-way ANOVA test. *p*-values from the analysis of variance were adjusted using the Holm approach to adjust for an inflated probability of a Type I error. When the ANOVA test revealed a statistical difference, pairwise comparisons were made by Tukey’s HSD (honestly significant difference) test. *p*-values < 0.005 were considered statistically significant. Statistical analysis was performed using the R statistical programming environment, version 3.5, RStudio Team (2020). RStudio: Integrated Development for R. RStudio, PBC, Boston, MA URL http://www.rstudio.com/.

## 5. Conclusions

Melasterol should be considered by clinicians in the tailored approach to the patients with acquired hypercholesterolemia who do not yet need pharmaceutical treatments or do not tolerate statins, or, finally, as supportive therapy. Besides its cholesterol-lowering effect, Melasterol is associated with a significant improvement in other relevant metabolic parameters such as body weight and blood glycemia. Noteworthy, the significative reduction in triglycerides levels could also have a positive impact on the prevention of cardiovascular risk. These results can be achieved with good feasibility and tolerability so that Melasterol can be considered a favorable option in the treatment of patients with impaired lipid profile.

## Figures and Tables

**Table 1:** Clinical characteristics of subjects enrolled in the study. Data were collected at baseline (T0) and after 3 months (T1) and 6 months (T2) of treatment and are expressed as mean ± SD.

				ANOVA		*t*-Test
	T0	T1	T2	F	*p*-Value	*p*-Value
Sex (m/f)	52/35					
Age (yrs)	58.3 ± 12.1					
Height (cm)	167.6 ± 8.5					
Weight (kg)	78.5 ± 15.9		76.3 ± 14.9			<0.001
BMI (kg/m^2^)	27.8 ± 4.4		27.0 ± 4.2			<0.001
Total cholesterol (mg/dL)	232.8 ± 21.3	203.2 ± 21.2	189 ± 19.4	91.99	<0.001	
HDL (mg/dL)	50.6 ± 21.4	49.4 ± 8.7	50.8 ± 9.1	0.2	0.8	
LDL (mg/dL)	141.8 ± 37.3	123.1 ± 28.1	114 ± 27.5	15.83	<0.001	
Triglycerides (mg/dL)	151.9 ± 37.3	128 ± 29.9	117.5 ± 23.9	25.1	<0.001	
Triglycerides/HDL	3.3 ± 1.13	2.7 ± 0.9	2.4 ± 0.8	16.6	<0.001	
Total cholesterol/HDL	5.0 ± 1.3	4.2 ± 0.9	3.9 ± 1.2	20.4	<0.001	
LDL/HDL	3.1 ± 1.1	2.6 ± 0.8	2.3 ± 0.9	12.01	<0.001	
Glycaemia (mg/dL)	108.3 ± 21.3		98.4 ± 18.6			<0.001

## Data Availability

Raw data are made available upon request to the corresponding author.
